# Peripubertal Exposure to Silver Nanoparticles (AgNPs) Triggers Follicle Activation, Probably Leading to “Burnout” by Stimulating the PI3K/AKT/mTOR/AMPK Signaling Pathway

**DOI:** 10.33549/physiolres.935737

**Published:** 2026-08-01

**Authors:** Saber NEHDI, Waleed ALDAHMASH, Abeer ALHAZMI, Anouar FERIANI, Maroua JALOULI, Awadh ALHELAISI, Jilani Purusottapatnam SHAIK, Saleh ALWASEL, Abdel Halim HARRATH

**Affiliations:** 1 Department of Zoology, College of Science, King Saud University, Riyadh, Saudi Arabia; 2 Department of Biology, College of Sciences, Umm Al-Qura University, Makkah, Saudi Arabia; 3 Laboratory of Biotechnology and Biomonitoring of the Environment and Oasis Ecosystems, University of Gafsa, Gafsa, Tunisia; 4 Department of Biology, College of Science, Imam Mohammad Ibn Saud Islamic University (IMSIU), Riyadh, Saudi Arabia; 5 Department of Biochemistry, College of Science, King Saud University, Riyadh, Saudi Arabia.

**Keywords:** Silver nanoparticles, Female fertility, Ovary, Folliculogenesis, Steroidogenesis, PI3K/AKT/mTOR pathway, AMPK

## Abstract

Despite the widespread use of silver nanoparticles (AgNPs) in consumer, industrial, and biomedical products, their potential effects on female reproductive health, particularly during critical developmental periods, remain insufficiently understood. The present study was therefore designed to investigate *in vivo* the dose-dependent effects of AgNP exposure on ovarian function and reproductive health in pubertal female rats. Pubertal Wistar albino female rats were divided into control and two AgNP groups (0.05 and 0.50 mg/kg/day). After one month of therapy, the females in the different groups were euthanized, and ovarian samples were collected. The results indicated that administration of AgNPs significantly increases the number of primordial follicles and significantly increases the number of growing follicles. Western blot analysis revealed that aromatase (Cyp19) and proliferating cell nuclear antigen (PCNA) were upregulated in both treatment groups. Similarly, the number of mRNA transcripts associated with folliculogenesis and steroidogenesis increased significantly in the high-dose treatment group. In addition, the level of growth differentiation factor 9 (GDF9) mRNA increased significantly in a dose-dependent manner. However, the levels of anti-Müllerian hormone (*Amh*) mRNA were considerably higher in the low-dose group and lower in the high-dose group, demonstrating that the detailed regulation of this gene is affected by AgNP dose. Analysis of the phosphatidylinositol 3-kinase signaling pathway revealed that compared to a 0.05 mg/kg, a AgNP exposure at 0.5 mg/kg significantly enhanced the PI3K/AKT/mTOR pathway and impacted *Ampk* signaling in a distinct manner. These findings suggest that AgNPs increase follicular development and deplete the follicular reserve, resulting in a phenomenon known as “burnout”.

## Introduction

The PI3K/AKT/mTOR signaling pathway is essential for regulating primordial follicle activation, granulosa cell proliferation, follicular growth, and the maintenance of ovarian reserve, whereas AMPK functions as a crucial cellular energy sensor that contributes to metabolic homeostasis, stress adaptation, and follicular survival [1]. The role of the PI3K/AKT/mTOR and AMPK signaling pathways in AgNP-induced ovarian dysfunction has not been adequately elucidated *in vivo*. Our prior research indicated that environmental toxicants could impair ovarian function by affecting folliculogenesis, steroidogenesis, autophagy, and apoptosis-related pathways [2–4]. Owing to their potential benefits to society, the use of silver nanoparticles (AgNPs) is increasing worldwide and has recently become one of the most often used forms of metal nanoparticles. [5]. They are used in consumer products such as food packaging [6], clothing and washing machines [7], pharmaceuticals [8], medical devices and bandages [9], and dental restoration materials [10], as well as being used in water treatment facilities [11]. Moreover, AgNPs have recently gained popularity because of their antifungal [12], antiangiogenic [13] and anti-cancer [14] properties, thereby leading to their extensive application in therapeutics. They are also used in the production of antibacterial products such as catheters or implants [15]. Specifically, there is a paucity of *in vivo* studies examining the impact of peripubertal exposure to AgNPs on primordial follicle activation, depletion of follicular reserve, and the molecular pathways governing folliculogenesis and steroidogenesis. Prior studies have predominantly concentrated on granulosa cell toxicity, oxidative stress responses, or *in vitro* reproductive models [16], but the mechanisms responsible for AgNP-induced ovarian “burnout” and premature reproductive age remain unclear.

The widespread use of AgNPs in consumer products has led to increased environmental and public health issues. [17]. Exposure to AgNPs has been shown to induce many cellular processes, such as mitochondrial dysfunction, DNA damage, apoptosis, and autophagy [18]. Many cellular signaling pathways can also be stimulated by AgNPs, including p42/p44, JNK, p38 mitogen-activated protein kinases and p53-dependent processes [19]. While the toxic effects of AgNPs on different tissues and organs have been well documented [20], their impact on mammalian ovaries remains limited [21] despite the involvement of this organ in determining the adult health of the new generation [22,23]. Notably, this fragile organ can be affected by environmental factors and diet, mainly in utero and in prepubertal stages, and is therefore indirectly responsible for the physical quality of offspring [2,24]. For example, exposure to brominated flame retardants (BFRs), which are included into several consumer products to reduce flame propagation, has been shown to decrease fertility in females [25]. BFRs have adverse effects on both folliculogenesis and steroidogenesis via the downregulation of expression of some ovarian genes, such as 17alpha-hydroxylase and insulin-like factor 3 [26]. In particular, BFRs have been reported to interfere with estrogen biosynthesis, granulosa cell function, and ovarian signaling pathways associated with follicle growth and reproductive hormone regulation [27]. These findings highlight the sensitivity of ovarian tissue to environmental toxicants and support the importance of investigating how emerging contaminants, including AgNPs, may similarly affect ovarian physiology and reproductive health.

Previous studies have established that the generation of reactive oxygen species (ROS) and inflammatory signaling are pivotal mechanisms contributing to the toxicity induced by AgNPs [28]. Exposure to AgNPs can elevate intracellular ROS levels, resulting in oxidative stress, mitochondrial impairment, DNA damage, apoptosis, and disrupted cellular metabolism [29]. Furthermore, excessive ROS production may trigger inflammatory signaling pathways, such as NF-κB and cytokine-mediated responses, thereby exacerbating cellular damage and tissue dysfunction [30]. The interplay between oxidative stress and inflammation is regarded as a significant factor in nanoparticle-induced toxicity across various organs, including reproductive tissues [31]. Nonetheless, the specific role of ROS-mediated inflammatory mechanisms in AgNP-induced ovarian dysfunction remains inadequately elucidated. The exact function of ROS-mediated inflammatory pathways in AgNP-induced ovarian dysfunction is not yet well understood.

The present investigation aimed to assess the impact of AgNP exposure during the peripubertal stage on the fertility of adult female rats by focusing on the ovaries. Despite numerous studies indicating that AgNPs can provoke oxidative stress, apoptosis, mitochondrial dysfunction, and steroidogenic impairment across various tissues and cell types, the specific impacts of AgNP exposure on ovarian follicular dynamics and reproductive lifespan are still poorly elucidated [32]. To accomplish this goal, ovarian function was evaluated by investigating the effects of AgNPs on the number of follicles, the cytoplasmic changes occurring within the oocyte, and the expression of crucial genes and proteins associated with folliculogenesis and steroidogenesis, with a specific emphasis on the PI3K/AKT/mTOR and AMPK signaling pathways. In fact, the PI3K/AKT/mTOR and AMPK signaling pathways play important roles in controlling cellular proliferation, metabolic activity, and viability [33]. Owing to their involvement in cellular responses to external stimuli such as nutrient availability, stress, and exposure to different agents such as nanoparticles [34], these pathways are frequently investigated in the context of cancer biology and metabolic diseases [35]. Nevertheless, the impact of various doses of AgNPs on vital cellular signaling pathways is still under investigation. However, it remains unknown whether exposure to AgNPs during the pivotal peripubertal phase induces analogous molecular and histopathological changes in ovarian tissue. Consequently, this study also explored to examine the dose-dependent effects of AgNPs on ovarian follicular development, ovarian reserve status, steroidogenesis-related markers, and the PI3K/AKT/ mTOR/AMPK signaling pathways in pubertal female rats, addressing a significant gap in the comprehension of AgNPs’ reproductive toxicity. We further elucidated that the work incorporates histological, ultrastructural, molecular, and signaling pathway investigations to furnish a comprehensive understanding of the impact of AgNPs on folliculogenesis and steroidogenesis throughout a pivotal developmental period. Our findings provide new mechanistic insights into the dose-dependent reproductive toxicity of AgNPs and their possible involvement in premature ovarian aging, reduced ovarian reserve, and reproductive diseases such as PCOS and early menopause.

## Materials and Methods

### Synthesis and characterization of the AgNPs

Silver nanoparticles were produced using a previously documented approach [cite our study IJN]. The metal salt precursor was an aqueous silver nitrate solution, with a sodium citrate solution acting asa stabilizing and reducing agent at elevated temperature. First, dissolve 0.001 M AgNO3 (99 %, Sigma Aldrich, USA) was dissolved in 50 mL of boiling water. Following that, 5 mL of 1 % trisodium citrate (99 %, Sigma Aldrich, USA) was added dropwise to the boiling solution. The colorless solution was agitated while boiling until a greenish-yellow color was obtained. The color change plainly indicated the creation of silver nanoparticles. At this point, the reaction was halted, the mixture was allowed to settle to room temperature, and the product was recovered using centrifugation. To remove excess silver ions, the silver colloids were washed at least three times with deionized (DI) water and dried at 70 °C for three consecutive days. A dried powder of nanoscale silver was obtained.

### Animal treatment

In this study, we used thirty female Wistar rats weighing between 200 and 250 g procured from the Animal Care Center. They were housed individually (22 °C to 24 °C) on a 12-hour light/12-hour dark cycle and given free access to food and water. The female rats were randomly assigned to three groups of ten each. Group 1 was the control group, and the rats were given 1 mL of distilled water via gavage. Group 2 rats were given 0.05 mg/kg b.w. of AgNPs orally every day. The rats in Group 3 were fed AgNPs orally at a dose of 0.5 mg/kg body weight per day. The selected doses were informed by prior toxicological and nanoparticle exposure research indicating biologically significant low- and moderate-dose impacts of AgNPs on mammalian organs, while avoiding severe systemic toxicity or mortality.

### Stereological study

Following 30 days of treatment, the animals were weighed and slaughtered. The ovaries were excised, weighed, and then fixed in neutral buffered formalin (NBF) for 24 hours. Histological sections were cut into 5-μm thick slices and stained with hematoxylin and eosin. The total number of follicles in each ovary was calculated using the approach published in our earlier study [31].

### Transmission electron microscopy (TEM)

The ovarian tissue was cut into 1 mm blocks for processing and frozen at 4 °C for 24 hours in 2.5 % glutaraldehyde in 0.1 M Na-cacodylate buffer (pH 7.2). Then, the tissue was fixed in 1 % osmium tetroxide for two hours. The samples were dehydrated with escalating ethanol concentrations and then embedded in resin. Semithin sections (0.5–1 μm) were prepared with the use of an ultramicrotome and glass knives (Leica Ultracut UCT, Australia) and stained with 1 % toluidine blue. An ultramicrotome and a diamond knife were used to cut ultrathin sections (50–80 nm). These sections were collected from the water surface onto 200G mesh copper wire grids and then allowed to dry on filter paper. To achieve high cell membrane contrast, the sections were first stained with 2 % uranyl acetate in 70 % alcohol for 10 minutes, followed by lead citrate.

Finally, the sections were rinsed with distilled water, dried on filter paper, and examined using a JEOL JEM 1011 transmission electron microscope (JEOL Ltd., Japan).

### Western blotting

In RIPA lysis buffer, which contained a protease inhibitor the homogenized ovarian tissues were lysed and the supernatants obtained after centrifugation were collected for Western blot analysis. The concentration of each protein was determined through the Bradford assay, separated using Mini-PROTEAN® TGX™ gels (Bio-Rad, Hercules, CA, USA) and transferred to PVDF membranes using a Trans-Blot Turbo Transfer System. The PVDF membranes were blocked with 5 % horse serum for 3 hours at room temperature before being incubated overnight at 4 °C with rabbit polyclonal antibodies against Cyp19 (diluted 1:200; ab184787, Abcam, Cambridge, UK) and PCNA (1:200, ab191606, Abcam, Cambridge, UK). The membranes were subsequently coated with secondary antibodies (1:500, sc-2357 and sc-516102; Sigma-Aldrich, St. Louis, MO, USA) for 2 hours at 37 °C. The target protein bands were photographed with a Bio-Rad Gel Documentation System and evaluated with Image Lab software (Bio-Rad, USA).

### qRT–PCR Assay and Gene expression analysis

RNA was extracted from ovarian samples using a RNeasy Mini Kit from Qiagen. The quality and integrity of the extracted RNA were assessed using a NanoDrop instrument on the basis of a 260/280 nm ratio. The RNA extracted was then reverse transcribed into cDNA using an iScriptTM cDNA synthesis kit (Applied Biosystems, Carlsbad, CA), following the manufacturer’s instructions. SYBR Green and gene-specific primers (Table 1) were used for real-time PCR on an Applied Biosystems 7500 Fast RT-PCR system (Carlsbad, CA). The protocol included an initial denaturation cycle at 95 °C for 2 min, followed by 40 cycles at 94 °C for 20 s, 58 °C for 20 s, and 72 °C for 20 s. The relative amount of each gene transcript was determined using the 2-DDCT method, with GAPDH serving as the reference gene for normalization.

### Statistics

All data are expressed as the means ± standard deviations (SDs). Using GraphPad Prism version 5 the statistical significance of differences in the mean values between groups was assumed for all p values (*p≤0.05, **p≤0.01, ***p≤0.001, ****p≤0.0001).

## Results

### Effects of AgNPs on changes in the number of follicles and oocyte ultrastructure

**Fig. 1:**
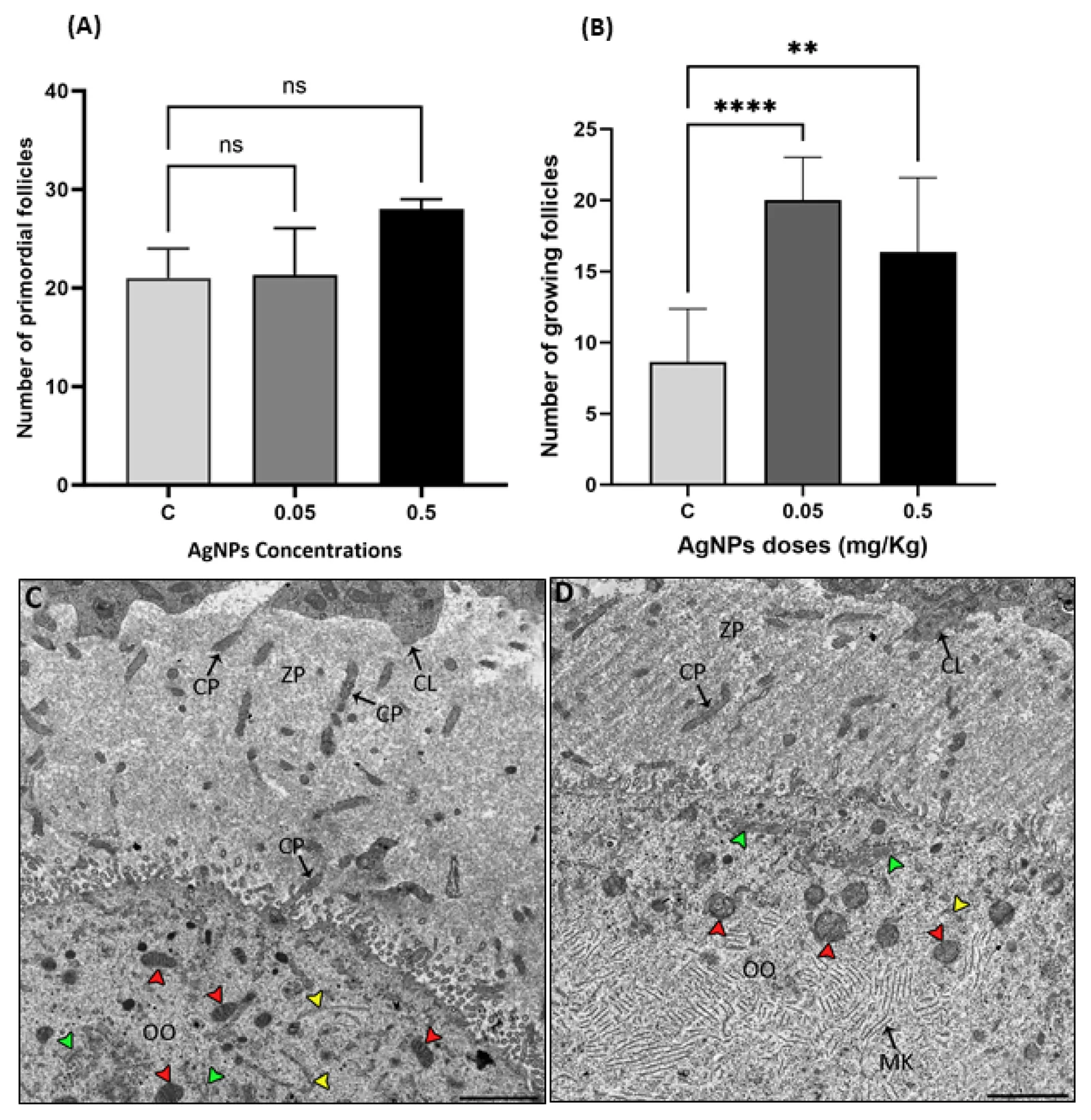
(A–B) Effects of AgNPs on the number of follicles in the treated groups compared with the control. No significant changes in the number of primordial follicles was found (**A**), whereas the number of growing follicles significantly increased (**B**). (**C–D**) Electron micrographs of oocytes from the treated groups compared with those from the control group. **C**) An oocyte (OO) from the control group showing classical normal cytoplasmic activities characterized by the presence of elongated mitochondria (red arrowheads), a Golgi complex (green arrowheads) and a rough endoplasmic reticulum (yellow arrowheads). The zona pellucida (ZP) is traversed by many cytoplasmic processes (CPs) from cumulus cells (CLs). **D**) An oocyte (OO) from the AgNP-treated group (0.5 mg/kg) presented high cytoplasmic activity compared with that of the control as the number and size of the Golgi complex (green arrowheads) increased, the mitochondria were larger and rounder (red arrowheads), and the membrane packets (MKs) that are distinctive of rat oocytes became very abundant.

As demonstrated in Fig. 1, both treatment groups had considerably more primordial follicles than the control group (Fig. 1). Additionally, the AgNP-treated groups had considerably more growing follicles than the control group (P < 0.05; Fig. 1B).

This finding implies that AgNPs may influence the initial pool of both primordial follicles and growing follicles.

Additionally, examination by electron microscope revealed that the oocytes from the control group mainly developed normal fine structures and classical cytoplasmic activities, which were characterized by the presence of elongated mitochondria, Golgi complexes and rough endoplasmic reticula (Fig. 1C). The zona pellucida is traversed by many cytoplasmic processes that originate from cumulus cells and terminate on the surface of the oocyte. However, compared with those from the control group, the oocytes from the AgNP-treated groups presented high cytoplasmic activity, as the number and size of the Golgi complex increased and the mitochondria were larger and rounder in shape (Fig. 1D). Moreover, the membrane packets that are distinctive to rat oocytes became very abundant. The observed modifications indicate that AgNPs have the potential to cause cellular stress or modify metabolic activity during oocyte development.

### Effect of AgNPs on Cyp19 and PCNA protein expression

**Fig. 2:**
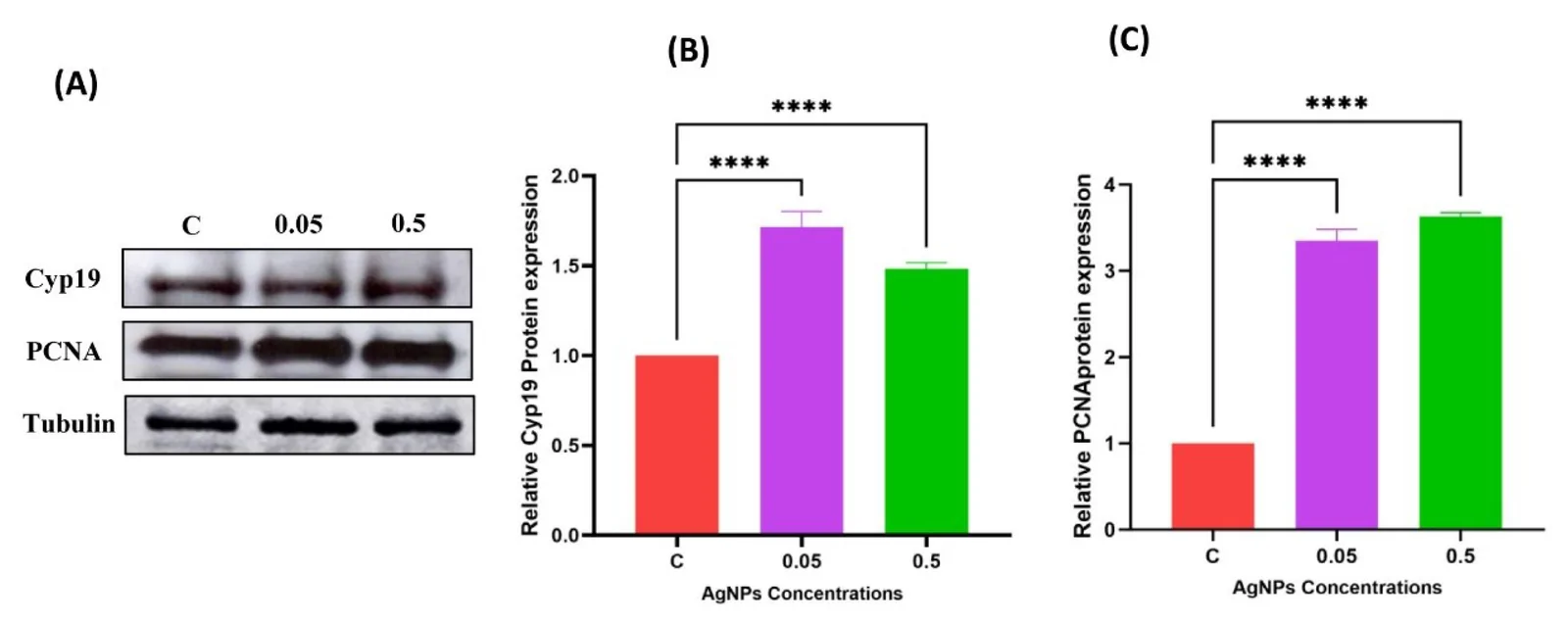
Effects of AgNPs on the protein expression levels of CYP19 and PCNA in the ovaries of rats in the treated groups (0.05 mg/kg and 0.5 mg/kg AgNPs) compared with those in the control group (**A–C**). Western blot analysis revealed that the protein expression levels of CYP19 and PCNA were significantly greater in both treatment groups than in the control group. (**B**) Relative CYP19 protein expression and (**C**) relative PCNA protein expression. All the data are expressed as the means ± SDs. * p < 0.05; ** p < 0.01; *** p < 0.001, p < 0.0001.

The results demonstrated that the levels of Cyp19 expression were markedly greater in the groups that received doses of 0.05 mg/kg and 0.5 mg/kg AgNPs than in the control group that did not receive drug treatment (Fig. 2A,B). These findings indicate that exposure to AgNPs may impact the mechanisms of estrogen production. Furthermore, when compared to the control group, the protein expression of PCNA, a biological marker of cell proliferation, was notably elevated in the study groups dosed with 0.05 mg/kg and 0.5 mg/kg AgNPs (Fig. 2A,C), suggesting an increase in cell proliferation. These results emphasize the possible biological impacts of AgNPs on protein expression associated with hormone function and cell differentiation.

### Effects of AgNPs on folliculogenesis- and steroidogenesis-related markers

**Fig. 3:**
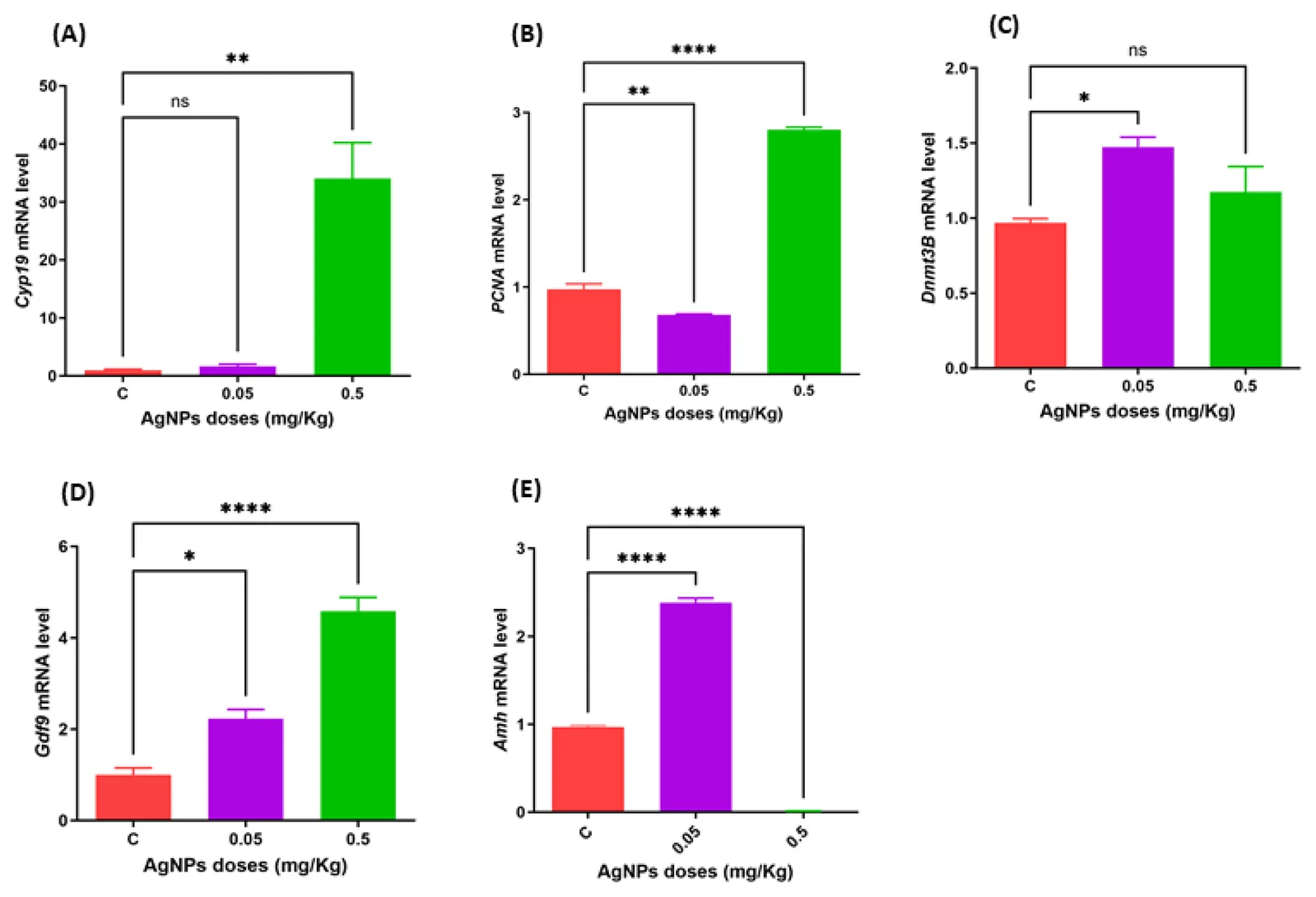
The mRNA expression levels of several folliculogenic and steroidogenic genes in the ovaries of the treated groups (0.05 mg/kg and 0.5 mg/kg AgNPs) were compared with those in the control group. **A**) CYP19 mRNA levels, (**B**) PCNA mRNA levels, (**C**) Gdf9 mRNA levels, (**D**) Gdf9 mRNA levels, and (**E**) Amh mRNA levels. All the data are expressed as the means ± SDs. * p < 0.05; ** p < 0.005; *** p < 0.001, p < 0.0001.

To assess the influence of AgNP exposure on ovarian function, the expression levels of many significant genes were examined. Compared to those in the control group, notable increases in the mRNA levels of Cyp19 were observed in the group treated with 0.5 mg/kg AgNPs (Fig. 3A). This observation implies that an increased dose of AgNPs could augment the enzymatic activity associated with the synthesis of estrogen. Furthermore, the dose-dependent response of PCNA was observed. The expression level of its mRNA markedly decreased in the ovaries that were administered a low dose of 0.05 mg/kg AgNPs but markedly increased in those exposed to the high dose of 0.5 mg/kg AgNPs (Fig. 3B). Although the group receiving 0.05 mg/kg AgNPs showed significant alterations in Dnmt3b gene expression (Fig. 3C), there were no significant differences in Dnmt3 mRNA levels between the high-dose group (0.5 mg/kg AgNPs) and the control group. Compared to the control group, both the low-dose and high-dose AgNP-treated groups had significantly higher mRNA levels of Gdf9, a key regulator of ovarian follicle growth (Fig. 3D).

However, mRNA levels of the anti-Müllerian hormone (Amh) gene, which controls follicle growth and development, increased significantly in the low-dose treatment group (0.05 mg/kg AgNPs) and dropped substantially in the high-dose group (0.5 mg/kg AgNPs) (Fig. 3E). This observation implies an intricate and dose-dependent reaction of Amh expression to AgNP exposure, which may have consequences on follicle reserves and reproduction longevity. Overall, the above results suggest that AgNPs have a selective effect on the expression of genes associated with folliculogenesis and steroidogenesis in the ovaries, which may affect the reproductive health of females.

### Effect of AgNPs on the AMPK/PI3K/AKT/mTOR/ signaling pathway

**Fig 4:**
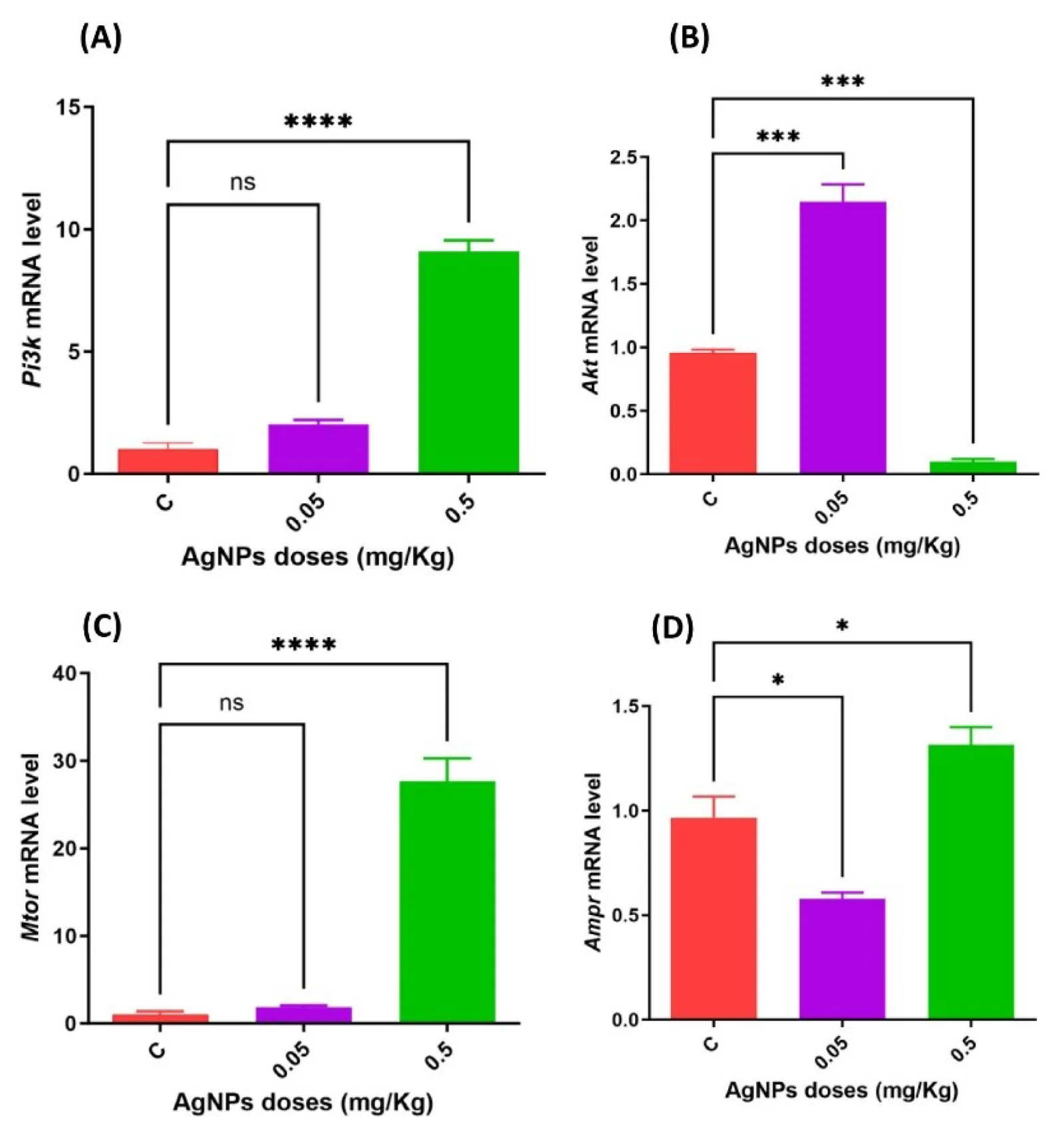
The mRNA expression levels of the genes encoding PI3K, AKT, mTOR and AMPK were detected by RT–PCR (A–D). (**A**) PI3K mRNA levels, (**B**) AKT mRNA levels, (**C**) mTOR mRNA levels, and (**D**) AMPK mRNA levels. All the data are expressed as the means ± SDs. * p < 0.05; ** p < 0.005; *** p < 0.0001; **** p < 0.00001.

An investigation of the expression levels of genes implicated in the AMPK/PI3K/AKT/mTOR signaling pathways demonstrated that the effects of AgNPs varied according to their dose. Administering a high dose of AgNPs (0.5 mg/kg) to rats resulted in a notable increase in the expression levels of the PI3K and mTOR genes (Fig. 4A,C). The observed phenomenon implies the activation of the PI3K/AKT/mTOR pathway, a well-recognized mechanism linked to cellular development and proliferation.

Notably, administering a low dose of AgNPs (0.05 mg/kg) did not significantly impact the expression of the PI3K and mTOR genes. This observation suggests the presence of a threshold effect, whereby only larger doses of AgNPs (> 0.05 mg/kg) stimulate this pathway. Conversely, the levels of AKT mRNA exhibited a clear trend; a notable increase was observed in the treated group with low dose of 0.05 mg/kg AgNPs, whereas a considerable decrease was recorded in the treated group with high dose of 0.5 mg/kg AgNPs (Fig. 4B). This finding indicates an intricate, dose-dependent regulation of the AKT component of the pathway, which may indicate distinct cellular stress reactions or adaptation processes at different levels of AgNPs. Conversely, a contrasting trend was observed when AMPK, a crucial energy sensor and regulator of cellular metabolism, was exposed to AgNPs. A significant decrease in AMPK mRNA levels was observed in the low-dose treatment group (0.05 mg/kg AgNPs), suggesting that the suppression of this system may impact cellular energy homeostasis and stress responses. Nevertheless, compared to the control group, the experimental group that received a greater dose of AgNPs (0.5 mg/kg) presented notable increases in AMPK mRNA levels (Fig. 4D).

## Discussion

Given the widespread usage of AgNPs in many consumer products and their potential impact on reproductive health, the effects of AgNPs on ovarian function and follicle growth have been a matter of substantial attention [36], especially when few studies have investigated them [21]. It is difficult to compare the biological activity of AgNPs across studies in terms of their physicochemical properties, such as their size, treatment efficacy, and applied AgNP dose because the species of animals used in each study vary considerably even if the target cells/tissues are the same; with that in mind, we did our best to investigate the effect of AgNPs on ovarian function [21].

Folliculogenesis and steroidogenesis are essential processes in the physiology of female reproduction, each with specific functions in the formation of ovarian follicle and the synthesis of sex hormones crucial for fertility and general reproductive well-being [4,37–39]. Comprehensive knowledge of the consequences of folliculogenesis and steroidogenesis is essential for the diagnosis and treatment of reproductive diseases, the enhancement of fertility therapies, and the mitigation of aging effects on female reproductive health [40, 41]. Our findings demonstrated that the number of developing follicles was notably greater in the AgNP treated groups than in the control groups, whereas the primordial follicle count was unchanged in all the groups. These findings suggest that AgNPs likely stimulate the activation and transition of dormant primordial follicles to primary and secondary follicles, leading to increased follicular development and depletion of the follicular reserve, a phenomenon known as “burnout” [42]. Our results are in agreement with those of previous studies showing that AgNPs induce oocyte maturation [43] but contradict the very few remaining studies showing that AgNPs have the potential to disrupt steroidogenesis [44] and the hormonal signaling pathways responsible for controlling follicular growth [45]. This difference in results may be due to the applied dose of AgNPs and the species of animal used in each study [21].

The ultrastructural examination of the oocytes revealed clear stimulation in response to AgNP treatment. Within the control group, the oocytes presented typical cytoplasmic anatomy, such as elongated mitochondria, a clearly defined Golgi complex, and a coarse endoplasmic reticulum. In contrast, oocytes from the group treated with AgNPs presented increased cytoplasmic activity, as evidenced by the presence of larger, rounder mitochondria and increased quantity and dimensions of the Golgi complex. Numerous scientific studies have revealed the relationship between mitochondrial conditions and biochemical and physiological details with pathophysiological importance, particularly the correlations with mitochondrial reactive oxygen species (mtROS) generation and mitochondrial calcium during various cellular perturbations [46]. In fact, in addition to their tubular form, swollen or elongated, mitochondria have also been shown to be “donut” shaped. This variation in mitochondrial shape can make this organelle adaptable in its role in both cellular survival and tissue homeostasis. It was proven that mitochondria are typically tubular under normal conditions, which is in agreement with our results, where the standard ooplasm exhibited elongated mitochondria [46]. Nevertheless, in the oocytes from the AgNP-treated group, the mitochondria started to become rounder (donut shaped). This mitochondrial shape has been previously interpreted as resulting from a slight increase in mtROS production, which controls many diverse cellular functions, such as cell proliferation, immunological functions, differentiation, and tissue remodeling [46, 47]. However, the blob design could be a beginning marker of irreversible toxicity. Therefore, the round-shaped mitochondria we found in the oocytes from the AgNP-treated group might indicate a slight increase in mtROS development induced by the presence of AgNPs, which induced both oocyte and granulosa cell proliferation and differentiation. In fact, ROS should be maintained at relatively low levels, which may communicate regular adjustments in the oxidative metabolic rate [47]; however, if the metabolic production of ROS exceeds the ability of endogenous antioxidant defense methods, oxidative stress can occur and lead to oxidative damage [47]. Prior research has shown that AgNPs can elevate intracellular ROS production, leading to mitochondrial dysfunction, lipid peroxidation, DNA damage, apoptosis, and the activation of inflammatory signaling [28]. Excessive ROS generation may disturb ovarian cellular homeostasis by impacting granulosa cell viability, steroidogenesis, follicular development, and mitochondrial integrity [48]. Furthermore, ROS-mediated activation of inflammatory pathways, such as NF-κB and pro-inflammatory cytokine signaling, may exacerbate ovarian tissue damage and reproductive dysfunction [49]. The increase in the cytoplasmic activity in the ooplasm of the oocytes from the treated groups, particularly the increase in the ER, may suggest that the AgNPs promoted folliculogenesis and steroidogenesis.

To confirm this hypothesis, we examined the influence of AgNPs on the expression of markers linked to ovarian function, including CYP19 and PCNA. The CYP19 gene, which encodes the biological enzyme aromatase and is thus alternatively referred to as the aromatase gene, is responsible for converting androgens into estrogens [50,51] and is crucial for the process of steroidogenesis [52]. On the other hand, PCNA is a protein that is closely linked to DNA replication and cell proliferation and is typically used as an indicator of cell proliferation [53]. Our RT–PCR analysis revealed that a high dose of AgNPs (0.5 mg/kg) resulted in elevated expression of CYP19 compared with the control, but a low dose did not (0.05 mg/kg). PCNA mRNA levels significantly decreased in the low dose group, while they increased in the high dose group. Western blot analysis revealed that the AgNP-treated groups exhibited a significant increase in the expression levels of both CYP19 and PCNA proteins when compared to the control group. Thus, these findings indicate that AgNPs may increase the synthesis of estrogen and the growth of granulosa cells, potentially impacting ovarian function and reproductive health. Furthermore, the increase in PCNA expression suggests an increase in cell proliferation, which may affect follicle growth and oocyte maturation. [54]. The expression levels of many genes associated with folliculogenesis and steroidogenesis, including Gdf9, Amh and Dnmt3B, have also been investigated to clarify the effects of AgNPs on ovarian function. Based on the TEM results, we found that exposure to AgNPs could enhance the expression of Gdf9 in a dose-dependent manner, which may impact the development of the oocyte. The modest alterations in Dnmt3B expression noted in the high-dose group indicate that the presence of AgNPs at this concentration may not have a significant effect on DNA methylation processes.

Nevertheless, the observed fluctuations in Amh expression are significantly higher in the group treated with low-dose and significantly lower in the group treated with high-dose highlight a complex pathophysiological and seemingly dose-dependent effects of AgNPs on follicular dynamics and ovarian function. Indeed, fluctuations (both increases and decreases) in AMH levels are associated with various pathological conditions, including polycystic ovary syndrome (PCOS) and early menopause [55]. The significantly increased mRNA expression levels of AMH in the low-dose group revealed the pathophysiologic effect of AgNPs on ovarian function. Low doses of AgNPs promoted folliculogenesis, as revealed by the significantly high expression of ovarian markers such as Cyp19, Gdf9 and PCNA. AMH is a glycoprotein hormone primarily produced by primary, preantral, and antral follicles, making it a reliable indicator of the ovarian reserve due to its stable levels throughout the menstrual cycle [55]. Thus, an increase in the number of growing follicles produces increased amounts of AMH, and elevated levels of AMH has been reported in patients with PCOS [56]. As a result, AMH has been investigated as a diagnostic marker for this female condition, either on its own or in conjunction with other markers, to enhance detection accuracy. [42]. However, the significantly lower levels of AMH in the high-dose group are another side of the coin with respect to the effect of AgNPs on ovarian function. Indeed, declining AMH levels have been linked to a gradual decrease in reproductive capacity with age, making it a potential indicator of early menopause. [55]. In fact, previous studies have shown that a decrease of 0.10 ng/mL in AMH is associated with a 14 % increased risk of early menopause (p < 0.001), Additionally, low AMH levels have been associated with an increased risk of miscarriage in infertile women who are anticipated to experience early menopause. [57]. Thus, AMH is clinically useful as a screening tool for diminished ovarian reserve [58]. It has also been reported that AMH levels can decrease due to anticancer drugs at any age [49]. Specifically, the alkylating agent cyclophosphamide, which is frequently used to treat various tumors and malignancies, is toxic to ovaries and can cause premature ovarian failure [59]. In agreement with our results, this chemical increases the activation of primordial follicles and increases the number of growing follicles, resulting in a “burnout” effect and subsequent loss of ovarian reserve [14]. Developing follicles, which are more sensitive to this anticancer drug, are damaged, resulting in decreased AMH production.

The PI3K/AKT/mTOR and AMPK signaling pathways are essential for controlling cellular growth, metabolism, and survival [60]. This study examined the impact of AgNPs on these pathways by analyzing the transcript levels of crucial genes in these processes. Our findings revealed that exposure to AgNPs led to dose-dependent alterations in gene expression. Specifically, a high dose (0.5 mg/kg AgNPs) increased the expression of PI3K and mTOR, whereas a low dose (0.05 mg/kg AgNPs) had no significant effect on their expression. Consistent with our results, Kalich-Philosoph *et al*. [59] reported that follicles from cyclophosphamide-treated mice presented increased levels of the phosphorylated forms of both PI3K and mTOR. In contrast, our analysis revealed that the levels of AKT mRNA increased in the low-dose group but decreased in the high-dose group, indicating that the intricate regulation of this pathway is dependent on the dose. Furthermore, AMPK expression exhibited divergent trends, characterized by decreased levels in the group receiving a low dose of AgNPs and increased levels in the group receiving a high dose of AgNPs. Previous findings indicate that AgNPs may regulate various signaling pathways in a dose-dependent manner, thereby impacting cellular metabolism and stress responses [21]. The stimulation of the PI3K/AKT/mTOR pathway by increased concentrations of AgNPs may have important consequences for cellular development and proliferation, considering the widely recognized functions of this pathway in these biological processes [61]. In contrast, the upregulation of AMPK expression at elevated levels could indicate a compensatory reaction to cellular stress, with the goal of restoring energy balance and enhancing cell survival [62]. We further acknowledged that mechanistic studies involving pathway-specific inhibitors, activators, or genetic modulation experiments are required to establish direct causal involvement of these signaling pathways.

The physicochemical properties of nanoparticles are essential factors influencing their biological activity, biodistribution, cellular uptake, and toxicological behavior [63]. The produced AgNPs were characterized by TEM examination, revealing primarily spherical nanoparticles with an estimated size range of 40–60 nm [64]. Furthermore, EDX analysis validated the elemental composition of silver in the produced nanoparticles [65]. Prior research indicates that nanoparticle dimensions, morphology, aggregation state, surface charge, and colloidal stability significantly impact their interactions with biological membranes and intracellular organelles, consequently influencing oxidative stress, inflammatory responses, apoptosis, and reproductive toxicity. Smaller nanoparticles typically exhibit a greater surface area-to-volume ratio, potentially enhancing cellular penetration and biological reactivity. Likewise, surface charge and zeta potential affect nanoparticle stability and their interactions with proteins and biological membranes [66]. This study primarily examined the biological and molecular effects of AgNP exposure on ovarian function; however, we recognize that further physicochemical characterization parameters, such as hydrodynamic diameter, zeta potential, and polydispersity index (PDI), would enhance the interpretation of the toxicological results. Consequently, further research should combine thorough nanoparticle characterization with mechanistic reproductive toxicology assessments to more effectively delineate the correlation between the physicochemical features of AgNPs and ovarian toxicity results. Moreover, the exposure conditions and dosages employed in laboratory settings may not accurately reflect chronic human environmental or occupational exposure situations. Consequently, prudence must be observed when assessing the possible clinical ramifications of AgNP exposure in human reproductive medicine. Further long-term investigations utilizing clinically pertinent exposure models, human ovarian tissues, and thorough endocrine and reproductive evaluations are essential to confirm the translational significance of these findings and to more accurately delineate the reproductive safety profile of AgNPs.

## Conclusions

The results of this current study provide important insights into the potential effects of AgNP exposure on ovarian function and reproductive health during the peripubertal period. The observed dose-dependent alterations in folliculogenesis, steroidogenesis-related markers, protein expression, and PI3K/AKT/mTOR/ AMPK signaling pathways highlight the complex relationship between AgNP exposure and ovarian physiology. Our findings indicate that AgNPs can stimulate follicular development and alter ovarian molecular signaling; however, prolonged or excessive follicular activation may adversely affect ovarian reserve maintenance. In particular, the observed changes in AMH expression suggest possible alterations in follicular dynamics and ovarian function, although these findings alone are insufficient to establish definitive diagnoses of reproductive disorders such as PCOS or premature ovarian insufficiency. Additional studies involving hormonal profiling, ovarian morphology analysis, reproductive outcome assessments, and long-term follow-up are required to clarify the clinical significance of these observations. Future investigations should also focus on the molecular mechanisms underlying AgNP-induced ovarian alterations and evaluate the long-term reproductive consequences of chronic exposure. Furthermore, comprehensive physicochemical character-rization and safety evaluation of AgNPs remain essential before their widespread biomedical application. Collectively, this study contributes novel mechanistic evidence regarding the reproductive toxicological effects of AgNPs and emphasizes the importance of considering dose-dependent biological responses when evaluating nanoparticle safety.

## Figures and Tables

**Table 1:** Primers for the real-time RT–PCR

Gene Symbol	Primer Seq
*Dnmt3A*	F: ACGCCAAAGAAGTGTCTGCT R: CTTGGCTATTCTGCCGTGTT
*Pcna*	F: CCTCACGACTTCATTGAGCA R: GGTAGCACACAGAGCGATGA
*Cyp19*	F: GGAGAATTCATGCGAGTCTGG R: TGCCGAATCGAGAGCTGTAA
*Gdf9*	F: GATGTGACCTCCCTCCTTCA R: GCCTGGGTACTCGTGTCATT
*GAPDH*	F: AGTTCAACGGCACAGTCAAG R: TACTCAGCACCAGCATCACC
*Pi3k*	F: AACTGAGTGCGTTCCAGGAG R: AGTTTTCTTTGCGCGTCGTA
*Amh*	F: AACTGAGTGCGTTCCAGGAG R: AGTTTTCTTTGCGCGTCGTA
*mTOR*	F: TGCCTTCACAGATACCCAGTAC R: AGGTAGACCTTAAACTCGGAC
*Ampk*	F: AGGAAGAATCCTGTGACAAGC R: CCGATCTCTGTGGAGTAGCAGT
